# Antibacterial activity of epigallocatechin gallate against *Trueperella pyogenes* and its potential effect on pyolysin

**DOI:** 10.3389/fvets.2026.1889070

**Published:** 2026-07-08

**Authors:** Guocheng Zhang, Xicai Liang, Jingyi Yang, Yueting Guo, Jia Chen, Ziyin Cui, Yunpu Wu, Yuru Guo, Zehui Zhang, Mingchun Liu

**Affiliations:** 1 College of Laboratory Animal Medicine, Liaoning University of Traditional Chinese Medicine, Shenyang, China; 2 School of Basic Medical Sciences, Liaoning University of Traditional Chinese Medicine, Shenyang, China; 3 College of Life Engineering, Shenyang Institute of Technology, Fushun, China; 4 Key Laboratory of Livestock Infectious Diseases in Northeast China, Ministry of Education, College of Animal Science and Veterinary Medicine, Shenyang Agricultural University, Shenyang, China

**Keywords:** antibacterial activity, epigallocatechin gallate, hemolytic activity, pyolysin, *Trueperella pyogenes*

## Abstract

*Trueperella pyogenes* (*T. pyogenes*) is widely associated with suppurative infections in domestic animals and causes substantial economic losses to livestock production. With the increasing incidence of these infections and the growing prevalence of antimicrobial resistance, alternative therapeutic strategies against *T. pyogenes* are urgently needed. This study demonstrated that epigallocatechin gallate (EGCG), a natural product from green tea, exhibited antibacterial activity against *T. pyogenes* (MIC = 16–64 μg/mL; MBC = 32–64 μg/mL). At subinhibitory concentrations, EGCG inhibited bacterial growth, disrupted cellular integrity, and suppressed biofilm formation in *T. pyogenes*. In a mouse model, EGCG attenuated *T. pyogenes* infection, reduced the bacterial load in the spleen, and mitigated splenic tissue damage. Transcriptional analysis showed that EGCG at 1/2 MIC downregulated the mRNA expression of the *plo* gene, which encodes pyolysin (PLO), in *T. pyogenes*. Moreover, EGCG inhibited the hemolytic activity of *T. pyogenes* ATCC 19411. Molecular docking and molecular dynamics simulations further indicated a stable interaction between EGCG and PLO. Our data demonstrate the efficacy of EGCG as a potential agent in combating *T. pyogenes* infections and provide preliminary insight into the potential mechanism for its inhibitory effect on PLO.

## 1 Introduction

*Trueperella pyogenes* (*T. pyogenes*) is a commensal, opportunistic, Gram-positive bacterium that causes purulent infections in domestic animals, including mastitis and endometritis in cattle, pneumonia and pleurisy in pigs, and lameness in goats (1–3). These infections result in substantial economic losses to the livestock industry (4). Some cases have highlighted the potential threat of *T. pyogenes*, which can be transmitted to humans through contact with domestic or wild animals (5, 6). A novel 23S ribosomal RNA methylase gene, *erm*(56), was recently identified in *T. pyogenes*. This gene imparts resistance to macrolide, lincosamide, and streptogramin B antibiotics in both *T. pyogenes* and *Escherichia coli* (7). The widespread resistance of *T. pyogenes* poses significant challenges to current treatments that predominantly rely on traditional antibiotics. Moreover, the barrier properties of biofilms significantly reduce the efficacy of antibiotics, leading to the persistence and recurrence of *T. pyogenes* infections (8). Therefore, it is imperative to investigate novel therapeutic agents against *T. pyogenes* infections.

The pathogenicity of *T. pyogenes* is primarily driven by its major virulence factors, particularly pyolysin (PLO), a member of the cholesterol-dependent cytolysin (CDC) family (9). Once secreted by *T. pyogenes*, PLO binds to cholesterol in the host cell membrane and assembles into transmembrane pores, causing membrane disruption and cell lysis (10). PLO-mediated cytotoxicity triggers intracellular K^+^ efflux in macrophages, which activates the NOD-like receptor family pyrin domain-containing 3 (NLRP3) inflammasome, leading to caspase-1 activation, gasdermin D (GSDMD) cleavage, pyroptosis, and IL-1β release (11, 12). Moreover, PLO from *T. pyogenes* has been reported to induce an immunosuppressive environment by triggering autophagy in endometrium stromal cells. This process facilitates invasion by other pathogens (13). Given its role in virulence and host immune regulation, PLO may be a potential target for anti-virulence strategies against *T. pyogenes* infections.

Epigallocatechin gallate (EGCG), one of the most abundant and bioactive flavonoids in green tea, has been widely reported to possess potent antimicrobial activity (14). EGCG inhibits various virulence factors in both Gram-positive and Gram-negative bacteria, including staphylococcal enterotoxin A (SEA), sortase A (SrtA) of *Streptococcus pneumoniae*, and quorum-sensing (QS)-regulated virulence factors of *Pseudomonas aeruginosa* (15–17). In addition, EGCG has been reported to reduce bacterial hemolytic activity by targeting hemolysins. EGCG attenuates staphylococcal alpha-hemolysin (Hla)-induced NLRP3 inflammasome activation and reduces hemolytic activity by inhibiting Hla secretion (18). Subinhibitory concentrations of EGCG inhibited the hemolytic activity of *Listeria monocytogenes*, and EGCG at 40 μg/mL significantly downregulated the transcription of the *hly* gene, which encodes hemolysin (19). These findings suggest that EGCG exhibits broad-spectrum antimicrobial activity by targeting multiple pathways.

However, the antibacterial effect of EGCG on *T. pyogenes* remains unexplored. In this study, we investigated the antibacterial activity of EGCG against *T. pyogenes*, focusing on its effects on bacterial cell structural integrity, biofilm formation, virulence, and hemolytic activity, and performed a preliminary analysis of its mechanism of action on PLO. Our data demonstrate the antibacterial and anti-hemolytic activities of EGCG against *T. pyogenes*, providing a potential basis for the development of new therapeutic agents against *T. pyogenes* infections.

## 2 Materials and methods

### 2.1 Ethics statement

Animal experiments in this study were conducted at Liaoning University of Traditional Chinese Medicine. All procedures were approved by the Laboratory Animal Welfare and Ethics Committee of Liaoning University of Traditional Chinese Medicine (No.: 21000062024173).

### 2.2 Strains and reagents

*Trueperella pyogenes* isolates (Supplementary Table 1) were collected from dairy cows and maintained at the Key Laboratory of Livestock Infectious Diseases in Northeast China, College of Animal Science and Veterinary Medicine, Shenyang Agricultural University. *T. pyogenes* ATCC 19411 was used as the reference strain. *T. pyogenes* strains were inoculated onto Mueller-Hinton agar (MHA; AOBOX, Beijing, China) supplemented with 5% (v/v) defibrinated sheep blood (Solarbio, Beijing, China) and cultured in Brain Heart Infusion (BHI) medium (AOBOX) containing 8% (v/v) fetal bovine serum (FBS; Cytiva, Marlborough, MA, USA). EGCG (purity ≥ 98%) and gentamicin sulfate (≥ 590 IU/mg) were purchased from Macklin Biochemical Co., Ltd. (Shanghai, China).

### 2.3 Antimicrobial susceptibility testing

The broth microdilution method was used to determine the minimum inhibitory concentration (MIC) of EGCG against *T. pyogenes*, according to Clinical and Laboratory Standards Institute guidelines (20). The lowest EGCG concentration that inhibited the growth of *T. pyogenes* was defined as the MIC. Bacterial viability was visualized using 0.1% (w/v) resazurin (Meilunbio, Dalian, China). For determination of the minimum bactericidal concentration (MBC), 100 μL aliquots from wells with no visible bacterial growth were subcultured onto MHA plates and incubated at 37 °C for 24 h. The MBC was defined as the lowest concentration at which no colonies were observed.

### 2.4 Growth curve

The effect of EGCG on *T. pyogenes* growth was evaluated by measuring growth curves as described previously (21). Suspensions of the ATCC 19411 strain were treated with different concentrations of EGCG (final concentrations of 1/8, 1/4, and 1/2 MIC) and cultured at 37 °C and 150 rpm. The OD_600 nm_ values were measured at various time points. Bacterial growth curves were plotted and analyzed after 30 h of incubation.

### 2.5 Determination of the bacterial activity

The viability of *T. pyogenes* ATCC 19411 was determined using the BacTiter-Lumi™ Luminescent Microbial Cell Viability Assay Kit (Beyotime, Shanghai, China). Bacterial cells were cultured to the logarithmic growth phase, harvested, and resuspended in PBS (pH 7.4) until the OD_600 nm_ of the suspension reached 1.0. The suspensions were treated with different EGCG concentrations and incubated at 37 °C for 30 min. Chemiluminescence was measured using a VICTOR Nivo Multimode Plate Reader (PerkinElmer, Waltham, MA, USA). The luminescence intensity was used as an indicator of relative bacterial viability.

### 2.6 Structural damage assessment of bacterial cells

#### 2.6.1 Cell wall damage assay

Bacterial cell wall integrity was assessed by measuring extracellular alkaline phosphatase (AKP) activity. The release of AKP from cells into the culture medium was measured as an indicator of cell wall damage, and the procedure was as follows. A bacterial suspension of ATCC 19411 was cultured to the logarithmic phase and diluted 100-fold in BHI medium supplemented with 8% fetal bovine serum. EGCG was added at final concentrations of 0, 1/8, 1/4, and 1/2 MIC, and the cultures were incubated at 37 °C with orbital shaking at 180 rpm for 24 h. The culture supernatants were collected, and AKP levels in bacterial cultures treated with different concentrations of EGCG were determined using an AKP assay kit (Jiancheng Bio, Nanjing, China).

N-phenyl-1-naphthylamine (NPN) is a hydrophobic fluorescent probe that fluoresces strongly in hydrophobic environments. Damage to the bacterial cell wall allows NPN to access the hydrophobic region of the cell membrane, thereby increasing fluorescence intensity. The effect of EGCG on the cell wall permeability of *T. pyogenes* was evaluated by measuring NPN fluorescence. *T. pyogenes* cells were prepared as described by Guo et al. (21), washed with PBS (pH 7.4), and standardized to equal cell density. The samples were divided into four groups and incubated with different concentrations of EGCG at 37 °C for 1 h. Following incubation, NPN was added to the bacterial suspension at a final concentration of 10 mM, and the suspension was incubated in the dark for 10 min. Fluorescence intensity was measured at excitation and emission wavelengths of 350 and 420 nm, respectively.

#### 2.6.2 Cell membrane damage assay

Extracellular β-galactosidase leakage was measured to assess changes in bacterial membrane permeability. *T. pyogenes* ATCC 19411 cells were cultured to the logarithmic growth phase in BHI supplemented with 2% lactose, harvested by centrifugation, washed with PBS (pH 7.4), and adjusted to an OD_600 nm_ of 0.2. The suspensions were divided into four groups. ONPG was added to a final concentration of 1.5 mM, followed by treatment with EGCG at final concentrations of 0, 1/8, 1/4, and 1/2 MIC. The bacterial suspensions were incubated at 37 °C for 100 min, and the OD_420 nm_ of the supernatant was measured at 20-min intervals to assess the leakage of intracellular β-galactosidase.

#### 2.6.3 Quantification of intracellular protein

*Trueperella pyogenes* ATCC 19411 was incubated with different concentrations of EGCG for 24 h as described in “Section 2.6.1.” Cells were then harvested by centrifugation, washed with PBS (pH 7.4), and resuspended to an OD_600nm_ value of 0.8. An aliquot (50 mL) of the bacterial suspension was collected and centrifuged to obtain a cell pellet. Total bacterial protein extraction reagent (APPLYGEN, Beijing, China) and protease inhibitors were added, and the total proteins were extracted by sonication. Protein concentration was determined using a bicinchoninic acid (BCA) assay. Protein samples were separated using sodium dodecyl sulfate-polyacrylamide gel electrophoresis (SDS-PAGE) with a 12% resolving gel. Equal volumes (50 μL) of each sample were loaded per lane. After electrophoresis, the gels were stained with Coomassie Brilliant Blue R-250 and visualized using a gel imaging system (Amersham ImageQuant 800; Cytiva).

#### 2.6.4 Quantification of intracellular nucleic acid

Intracellular nucleic acids in *T. pyogenes* were relatively quantified by measuring the fluorescence intensity of 2-(4-Amidinophenyl)-6-indolecarbamidine dihydrochloride (DAPI) (Solarbio). *T. pyogenes* ATCC 19411 was treated with different concentrations of EGCG as described in “Section 2.6.1.” Cells were harvested and resuspended in PBS (pH 7.4) to an OD_600 nm_ value of 0.8. The cell suspension was then incubated with DAPI for 30 min. The fluorescence intensity was measured at an excitation wavelength of 364 nm and an emission wavelength of 454 nm.

### 2.7 Biofilm susceptibility test

The minimum biofilm inhibitory concentration (MBIC) and minimum biofilm eradication concentration (MBEC) were determined according to a previous study (22). Briefly, EGCG at different concentrations was incubated with bacterial suspensions (10^5^ CFU/mL) and added to the wells of a 96-well plate (200 μL per well). After 48 h of incubation at 37 °C for biofilm formation, the supernatant was carefully removed, and the wells were washed three times with PBS (pH 7.4). Biofilm formation was quantified using crystal violet staining, and the absorbance was measured at 595 nm. Biofilm biomass was calculated relative to the untreated control after subtracting the OD_595 nm_ values of the blank wells. The MBIC was defined as the lowest concentration that reduced the biofilm biomass by at least 90%.

For measuring the MBEC, biofilms were first allowed to mature before exposure to different concentrations of EGCG. Following treatment, the biofilms were suspended, and the number of viable bacteria was determined by plate counting. The MBEC was defined as the lowest concentration at which no viable cells were recovered from preformed biofilms.

### 2.8 Biofilm observation

The effects of EGCG on *T. pyogenes* biofilm formation were examined using confocal laser scanning microscopy (CLSM). One milliliter of *T. pyogenes* suspension (1 × 10^5^ CFU/mL) was added to glass-bottom cell culture dishes (NEST, Wuxi, China), followed by treatment with EGCG at final concentrations of 1/2 MIC, 1 MIC, and 2 MIC. After 48 h of incubation at 37 °C, the culture medium was removed, and the samples were washed twice with PBS (pH 7.4). The samples were stained with SYTO9 and propidium iodide (PI) using the LIVE/DEAD BacLight Bacterial Viability Kit (Invitrogen, Camarillo, CA, USA) for 25 min, washed twice with PBS (pH 7.4), and examined using a confocal microscope (Stellaris, Leica, Germany) to evaluate the biofilm morphology. SYTO9 labels intracellular DNA in intact cells, while PI binds to DNA only in membrane-compromised cells.

### 2.9 Animal experiment

Female Kunming mice (6–8 weeks of age) were purchased from AOJIE Biological Technology Co., Ltd. (Shenyang, China; Permit No.: SCXK (Liao) 2023–0004) and kept under specific pathogen-free conditions. The mice were maintained on a 12-h light/12-h dark cycle and had unlimited access to food and water. In the survival experiment, mice were acclimatized for 1 week and then randomly divided into six groups (*n* = 6 per group): control group, model group, low-dose EGCG group (12.5 mg/kg b.w. per day), medium-dose EGCG group (25 mg/kg b.w. per day), high-dose EGCG group (50 mg/kg b.w. per day), and positive control group (gentamicin, 4 mg/kg b.w. per day). Gentamicin and EGCG were administered intraperitoneally (0.1 mL/10 g), whereas the control and model groups received PBS (pH 7.4; 0.1 mL/10 g). The drug was administered once daily for 3 days. Subsequently, mice in each group were intraperitoneally challenged with 1 × 10^9^ CFU of *T. pyogenes*, and the control group received an equal volume of PBS. The animals were observed for 5 days, and survival was recorded every 12 h.

Mice were randomly divided into four groups, with six mice in each group, to evaluate the effect of EGCG on *T. pyogenes*-induced infections. The four groups included the control group, model group, EGCG-treated group (50 mg/kg b.w. per day), and positive control group (gentamicin; 4 mg/kg b.w. per day). All mice were intraperitoneally infected with 2 × 10^8^ CFU of *T. pyogenes* ATCC 19411, and the control group received an equal volume of PBS. Two hours later, mice in the EGCG and gentamicin groups received intraperitoneal injections (0.1 mL/10 g), whereas the control and model groups received an equal volume of PBS. The treatments were administered once daily for three consecutive days. After 72 h, the mice were euthanized, and their spleens were collected to determine the spleen index as previously described by Murshed et al. (23). Equal amounts of spleen tissue were homogenized in 1 mL of sterile PBS. The homogenates were serially diluted with sterile PBS and plated on MHA supplemented with 5% defibrinated sheep blood and 0.1% colistin sulfate salt to determine the bacterial load in the spleen. Spleen tissues from each group of mice were subjected to histopathological analysis to assess infection severity, and representative images were captured at 100 × magnification.

### 2.10 Transcriptome analysis

The suspension of the ATCC 19411 strain was diluted 1:100 (v/v) in BHI medium and treated with 1/2 MIC of EGCG. A control sample without EGCG was also prepared for comparison. The suspensions were incubated at 37 °C with shaking at 150 rpm. When the culture reached the late-log phase (OD_600 nm_ = 1.0), cells were sampled, and total RNAs were extracted using FreeZol Reagent (Vazyme, Nanjing, China). Total RNAs were sent to Sangon Biotech Co., Ltd. (Shanghai, China) for RNA-seq analyses. Bioinformatics analyses were performed by Sangon Biotech. The published genome sequence of *T. pyogenes* TP8 (GenBank accession NO.: CP007003) was used as a reference for transcriptomic analysis (24). Genes were considered differentially expressed when the log2 fold change value was more than 1 or less than −1 and the q-value was less than 0.05.

Ten differentially expressed genes (DEGs) were selected to validate transcriptome results. Total RNAs extracted as described above were subjected to reverse transcription using the PrimeScript RT reagent Kit (TaKaRa, Dalian, China). Real-time fluorescence quantitative PCR (qPCR) was performed to compare the mRNA expression levels in ATCC19411 after treatment with EGCG using the Taq Pro Universal SYBR qPCR Master Mix (Vazyme). The cDNA samples were diluted and used as templates for qPCR. *16S rRNA* was used as a reference gene, and the primer sequences used for qPCR are shown in Supplementary Table 2. Amplification and quantification were performed using a qTOWER3 thermal cycler (Analytik Jena GmbH, Jena, Germany). The data were analyzed using the qPCRsoft software provided with this instrument.

### 2.11 Analysis of *plo* gene expression

*Trueperella pyogenes* strains isolated from dairy cows were treated with EGCG at 1/2 MIC. The relative expression of *plo* was quantified by qPCR as described in “Section 2.10,” and fold changes were calculated using the 2^−ΔΔCt^ method.

### 2.12 Assessment of the hemolytic activity of *Trueperella pyogenes*

A logarithmic phase *T. pyogenes* ATCC 19411 suspension was diluted 1:100 (v/v) and treated with EGCG at 0, 1/8, 1/4, and 1/2 MIC. The suspensions were incubated at 37 °C with shaking (150 rpm) to an OD_600 nm_ value of 1.5, followed by centrifugation at 10,000 × *g* for 5 min to collect the supernatants. Bacterial culture supernatant (100 μL) was mixed with 875 μL of PBS (pH 7.4) and 25 μL of defibrinated sheep blood. The mixture was incubated at 37 °C for 1 h and centrifuged at 4,000 × *g* for 2 min. The resulting supernatant was collected, and the absorbance was measured at 543 nm. PBS and 1% Triton X-100 were used as negative and positive controls, respectively, instead of the supernatant. The hemolytic activity of one sample from the untreated control group was set to 100%, and the relative hemolytic activity was calculated for each group.

### 2.13 Molecular docking

The chemical structure of EGCG was obtained from the PubChem database (CID: 65064; https://pubchem.ncbi.nlm.nih.gov), and the crystal structure of PLO was retrieved from the AlphaFold Protein Structure Database (AlphaFold DB; model ID: AF-O31241-F1; https://alphafold.ebi.ac.uk). The PLO structure was prepared using PyMOL by removing water molecules and other heteroatoms. The structures were further processed using AutoDock Tools by adding polar hydrogen atoms and assigning the Gasteiger charges. Molecular docking was performed using AutoDock Vina to evaluate the interaction between EGCG and PLO. The optimal binding conformation was selected based on the predicted binding energy. The interactions between EGCG and amino acid residues of PLO were visualized using Discovery Studio 2019 and PyMOL, and two-dimensional interaction diagrams and three-dimensional structures were generated.

### 2.14 Molecular dynamics simulations

Molecular dynamics simulations of the EGCG–PLO complex were performed using GROMACS 2022.3 software. The AMBER14SB force field was used for the receptor protein, and the topology of EGCG was generated with sobtop_1.0 (dev3.1) using GAFF2 parameters and RESP charges. The system was placed in a cubic box and solvated with the TIP3P water model. Na^+^ and Cl^−^ ions were then added to neutralize the system and to obtain a final salt concentration of 0.15 M NaCl. Long-range electrostatic interactions were handled by the particle mesh Ewald (PME) method, and bond constraints were applied using the LINCS algorithm. After energy minimization, a 100 ns simulation was run under the NPT ensemble at 310 K and 1 bar, with a time step of 2 fs. The trajectory was analyzed using GROMACS tools to calculate the root-mean-square deviation (RMSD), root-mean-square fluctuation (RMSF), number of hydrogen bonds, radius of gyration (Rg), and solvent-accessible surface area (SASA). The free energy landscape (FEL) was constructed using RMSD and Rg as reaction coordinates to evaluate the conformational stability of the complex.

### 2.15 Statistical analysis

All *in vitro* assays were performed with three replicates, while animal experiments included six mice per group. Data are presented as the mean ± standard deviation (SD). Statistical analyses were performed using GraphPad Prism version 8.0 (GraphPad Software, San Diego, CA, USA). *p-*values were calculated using either a one-way analysis of variance among multiple groups or an unpaired two-tailed Student’s t-test between two groups. Statistical significance was defined as *p* < 0.05 and *p* < 0.01.

## 3 Results

### 3.1 Antibacterial activity of EGCG against *Trueperella pyogenes*

**Figure 1:**
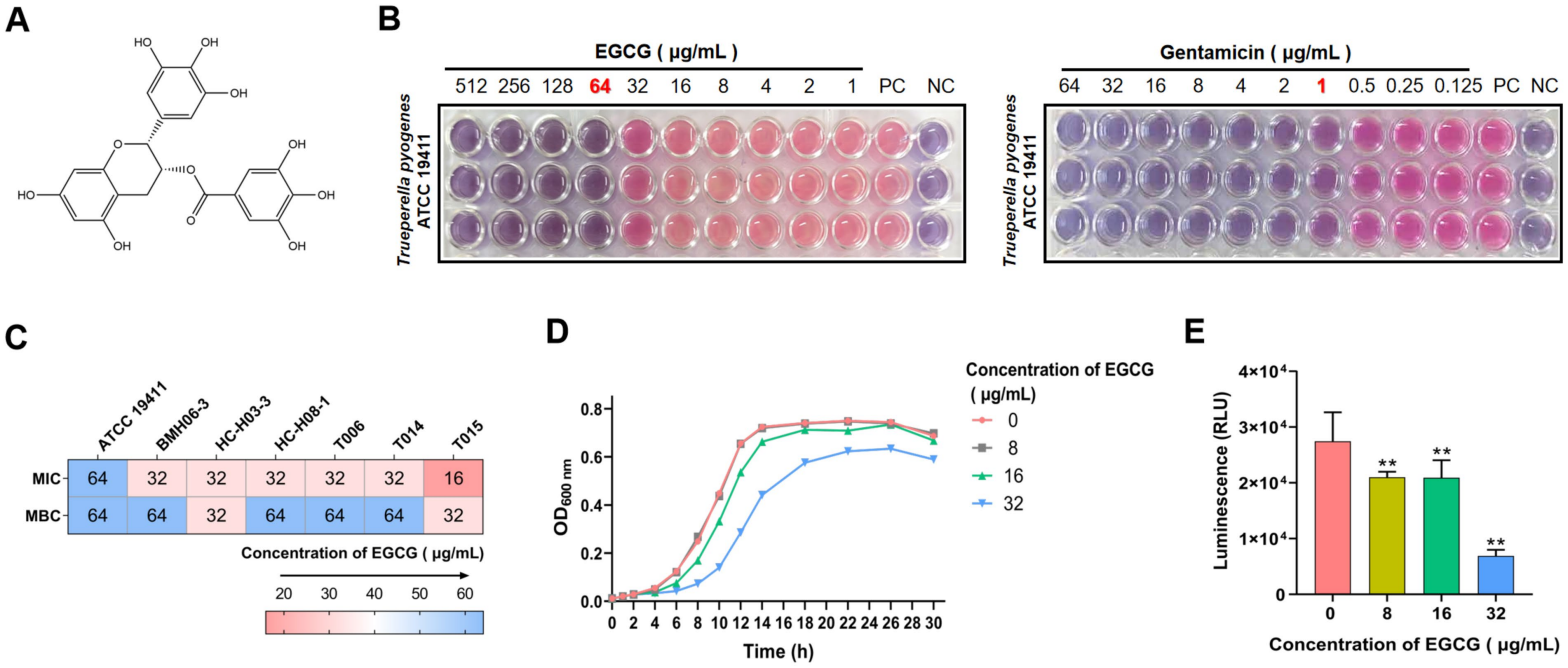
*In vitro* antibacterial activity of EGCG against *T. pyogenes*. **(A)** Chemical structure of EGCG. **(B)** Determination of the MICs of EGCG and gentamicin against *T. pyogenes* ATCC 19411. PC, positive control; NC, negative control. **(C)** The activity of EGCG against *T. pyogenes* ATCC 19411 and bovine isolates. **(D)** Growth curve of ATCC 19411 treated with different concentrations of EGCG. **(E)** Viability of ATCC 19411 cells after EGCG treatment. Statistical significance compared with the control group (untreated with EGCG) is indicated as *p < 0.05 and **p < 0.01. Data are presented as mean ± SD from three replicates.

The chemical structure of EGCG is shown in Figure 1A. To evaluate its antibacterial activity against *T. pyogenes*, we determined the MICs and MBCs against the reference strain ATCC 19411 and six bovine isolates. The MICs of EGCG and gentamicin against ATCC 19411 were 64 μg/mL and 1 μg/mL, respectively (Figure 1B). Among the tested strains, the MICs of EGCG ranged from 16 to 64 μg/mL, and the MBCs ranged from 32 to 64 μg/mL (Figure 1C).

The growth curves of ATCC 19411 treated with different concentrations of EGCG are shown in Figure 1D. EGCG at 1/8 MIC (8 μg/mL) had no obvious effect on bacterial growth, whereas EGCG at 1/4 MIC (16 μg/mL) and 1/2 MIC (32 μg/mL) prolonged the logarithmic phase, delayed entry into the stationary phase, and inhibited bacterial growth. Bacterial viability was assessed using an ATP-dependent luciferin-luciferase luminescence assay, and luminescence intensity was used as an indicator of cell viability. EGCG at subinhibitory concentrations significantly reduced the viability of ATCC 19411, with the strongest effect observed at 1/2 MIC (Figure 1E). These results indicate that EGCG exhibits antibacterial activity against *T. pyogenes in vitro*.

### 3.2 Effects of EGCG on bacterial cell structure

**Figure 2:**
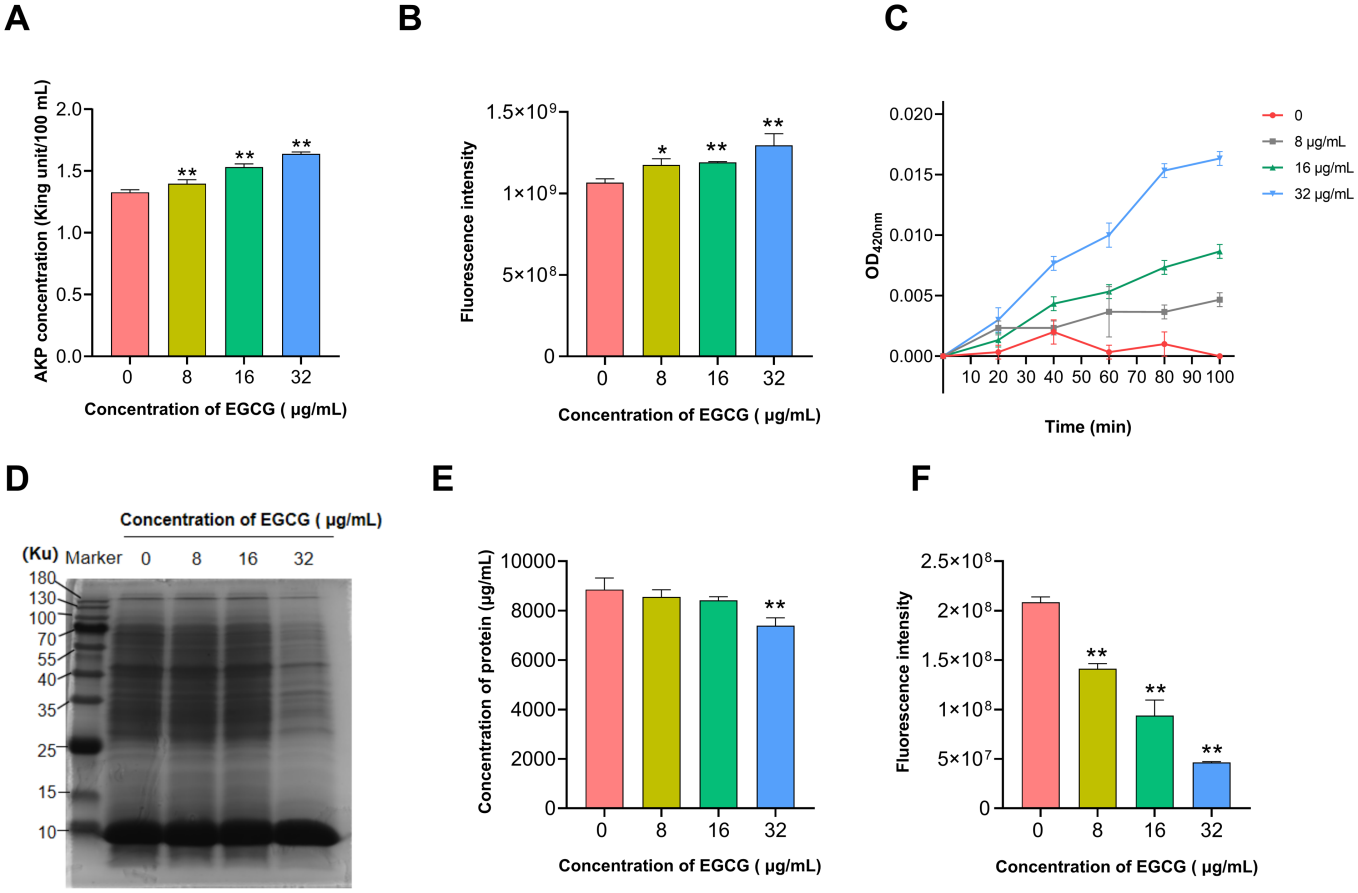
Effects of subinhibitory concentrations of EGCG on the cell integrity of *T. pyogenes*. **(A)** Leakage of AKP from ATCC 19411. **(B)** NPN uptake by ATCC 19411. **(C)** Leakage of *β*-galactosidase from ATCC 19411. **(D)** SDS-PAGE analysis of intracellular protein profiles. **(E)** Intracellular protein content determined by the BCA assay. **(F)** Intracellular nucleic acid content. Statistical significance compared with the control group (untreated with EGCG) is indicated as *p < 0.05 and **p < 0.01. Data are presented as mean ± SD from three replicates.

To further examine the effects of EGCG on the cellular integrity of *T. pyogenes*, changes in the cell wall, cell membrane, intracellular proteins, and nucleic acids were assessed after EGCG treatment. The cell wall and cell membrane are essential for maintaining cellular structural integrity and preventing the leakage of intracellular contents. As shown in Figure 2A, EGCG at subinhibitory concentrations significantly increased AKP levels in the culture supernatant of *T. pyogenes* in a concentration-dependent manner. The release of AKP into the extracellular medium suggests that EGCG disrupted cell wall integrity. EGCG treatment at subinhibitory concentrations significantly increased NPN fluorescence intensity in *T. pyogenes*, indicating disruption of cell wall integrity and enhanced NPN uptake (Figure 2B). Damage to the bacterial cell membrane was examined by monitoring ONPG hydrolysis. Following ONPG addition, the EGCG-treated samples showed a gradual increase in absorbance at 420 nm, and a concentration-dependent response to EGCG was observed after 60 min (Figure 2C). This increase was consistent with greater membrane permeability in *T. pyogenes*, allowing ONPG to enter the cells and be hydrolyzed by intracellular *β*-galactosidase to o-nitrophenol.

Proteins and nucleic acids are major intracellular components of bacterial cells; therefore, the effects of EGCG on these components were then examined. SDS-PAGE analysis showed that most protein bands from *T. pyogenes* treated with EGCG at 1/2 MIC were markedly reduced in intensity (Figure 2D). Total cellular protein content was further quantified using the BCA assay. Compared with the untreated control, EGCG at 1/8, 1/4, and 1/2 MIC reduced total cellular protein content by 3.30, 4.87, and 16.47%, respectively (Figure 2E). DAPI staining revealed that intracellular nucleic acid levels were significantly reduced after treatment with subinhibitory concentrations of EGCG. At 1/8, 1/4, and 1/2 MIC, the fluorescence intensity of intracellular nucleic acids decreased by 32.25, 54.95, and 77.76%, respectively (Figure 2F). EGCG therefore compromised the cell wall and bacterial cell membrane integrity of *T. pyogenes* and altered intracellular protein and nucleic acid levels.

### 3.3 Effects of EGCG on biofilm formation of *Trueperella pyogenes*

**Figure 3:**
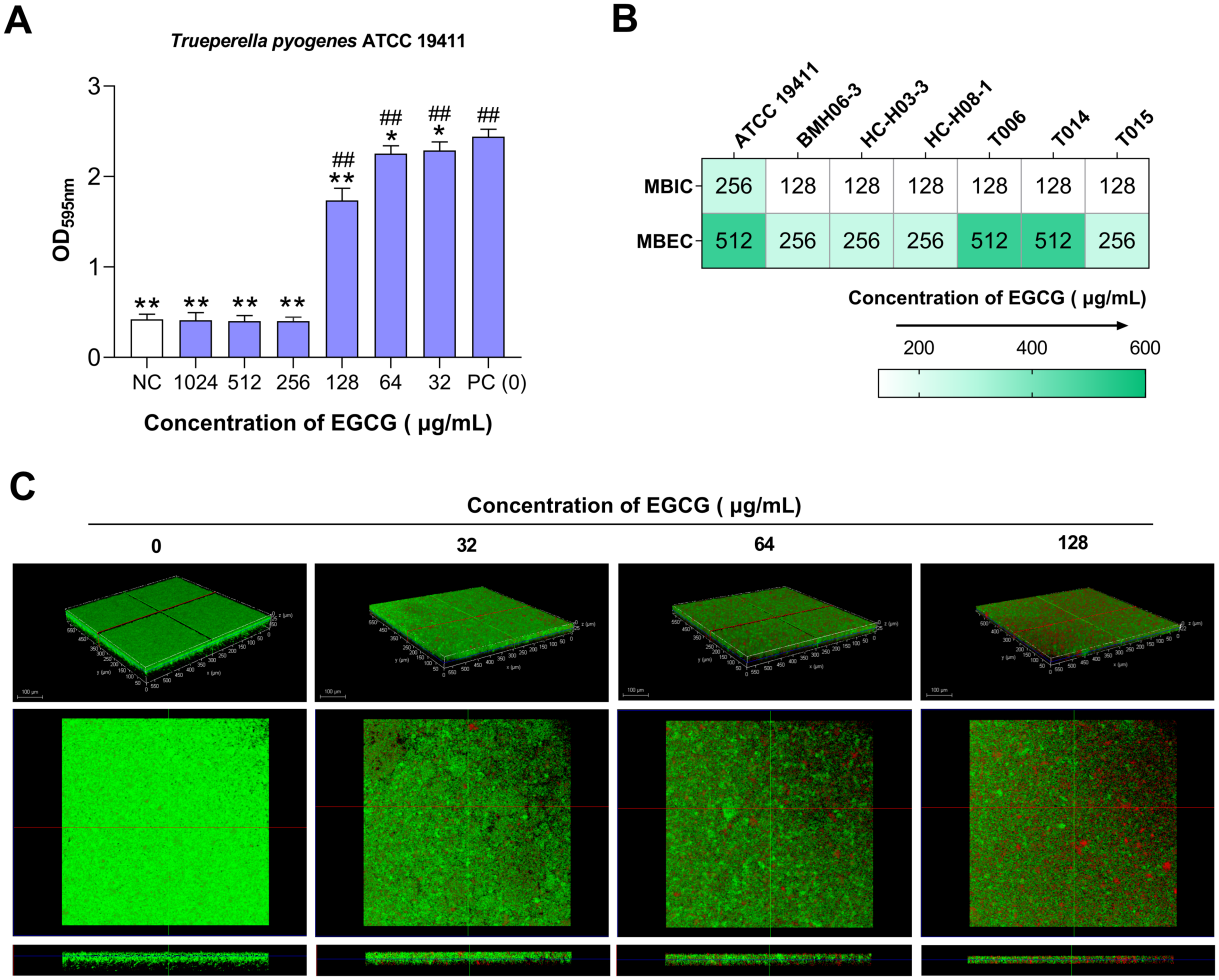
Antibiofilm activity of EGCG against *T. pyogenes*. **(A)** Effect of EGCG on biofilm formation by *T. pyogenes* ATCC 19411 determined by crystal violet staining. **(B)** MBIC and MBEC of EGCG against *T. pyogenes*. **(C)** CLSM analysis of the inhibitory effect of EGCG on biofilm formation by ATCC 19411. *p < 0.05 and **p < 0.01, compared with the positive control (PC); ^#^*p* < 0.05 and ^##^*p* < 0.01, compared with the negative control (NC). Data are presented as mean ± SD from three replicates.

Biofilm susceptibility assays showed that EGCG effectively inhibited biofilm formation by *T. pyogenes*. Crystal violet staining indicated that EGCG at concentrations ≥256 μg/mL reduced the biofilm biomass of ATCC 19411 by more than 90% (Figure 3A). The MBICs of EGCG against *T. pyogenes* ranged from 128 to 256 μg/mL, whereas the MBECs ranged from 256 to 512 μg/mL (Figure 3B). As shown in Figure 3C, CLSM images of SYTO9/PI-stained biofilms displayed green fluorescence from viable bacteria and red fluorescence from dead bacteria. Compared with the control group, EGCG at 1/2 MIC, MIC, and 2 MIC reduced biofilm formation by ATCC 19411. EGCG disrupted the dense biofilm structure of *T. pyogenes*, resulting in a looser and flatter architecture. After treatment with EGCG at 2 MIC (128 μg/mL), the biofilm became thinner and contained more dead bacteria.

### 3.4 EGCG attenuates the virulence of *Trueperella pyogenes* in a murine infection model

**Figure 4:**
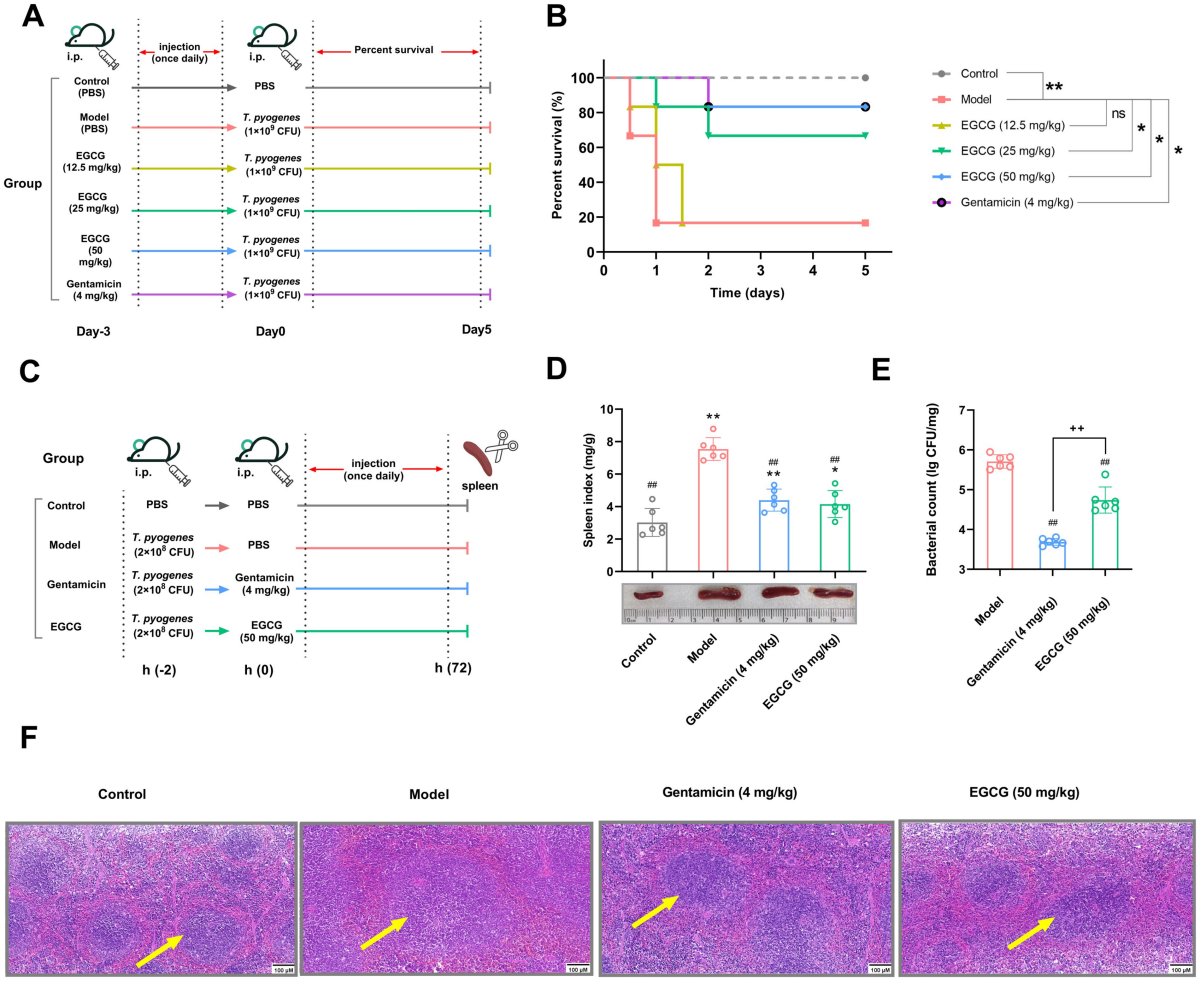
Protective and therapeutic effects of EGCG against *T. pyogenes* infection in mice. **(A)** Schematic overview of the experimental protocol for evaluating the protective effect of EGCG. **(B)** Survival rates of mice infected with *T. pyogenes* ATCC 19411. **(C)** Experimental design for assessing the therapeutic effect of EGCG. **(D)** Spleen index of mice in different groups. **(E)** Splenic bacterial load in different groups. **(F)** Histopathological evaluation of spleens in different groups. *p < 0.05 and **p < 0.01, compared with the control group; ^#^*p* < 0.05 and ^##^*p* < 0.01, compared with the model group; ^+^*p* < 0.05 and ^++^*p* < 0.01, compared with the gentamicin group; ns, not significant. Data are presented as mean ± SD; n = 6 per group.

A murine infection model was used to determine whether EGCG could attenuate *T. pyogenes* virulence *in vivo*. The experimental design is shown in Figure 4A, and the survival results are presented in Figure 4B. After challenge with ATCC 19411, 83.33% of untreated mice died within 24 h. Administration of EGCG at 50 mg/kg or gentamicin at 4 mg/kg significantly improved survival (*p* < 0.05), with mortality reduced to 16.67%. A 25 mg/kg dose of EGCG lowered mortality to 33.33%, whereas 12.5 mg/kg only slightly prolonged survival time in infected mice (Figure 4B). These results suggest that EGCG (50 mg/kg) exerts a protective effect against *T. pyogenes* infection in mice.

The therapeutic effect of EGCG against *T. pyogenes* infection was evaluated using an intraperitoneal infection model, as shown in Figure 4C. Intraperitoneal infection caused splenic enlargement in mice, which was reflected by an increased spleen index. Treatment with EGCG (50 mg/kg) or gentamicin (4 mg/kg) alleviated splenomegaly and reduced the spleen index (Figure 4D). Compared with the model group, treatment with EGCG at 50 mg/kg reduced the bacterial load in the spleen by more than 7-fold, while gentamicin at 4 mg/kg produced a further reduction (Figure 4E). Hematoxylin and eosin (H&E) staining at 100 × magnification showed expansion of the splenic white pulp in the model group. This pathological change was less pronounced in mice treated with EGCG or gentamicin (Figure 4F). EGCG exhibited *in vivo* activity against *T. pyogenes* and may alleviate infections caused by this bacterium.

### 3.5 Effect of EGCG on *plo* expression in *Trueperella pyogenes*

Transcriptomic analysis was performed to identify DEGs in ATCC 19411 cells treated with EGCG at 1/2 MIC for 24 h. A total of 247 DEGs were identified using the criteria of q-value < 0.05 and |log2 fold change| > 1 (Supplementary Figure 1). KEGG pathway analysis showed that bacterial virulence-related pathways (two-component systems and QS system) were downregulated (Supplementary Figure 2). The qPCR data for the 10 selected DEGs are shown in Supplementary Figure 3, and the changes in mRNA expression levels were consistent with the transcriptomic results. Among the DEGs, the QS system-associated *plo* gene was downregulated after EGCG treatment.

**Figure 5:**
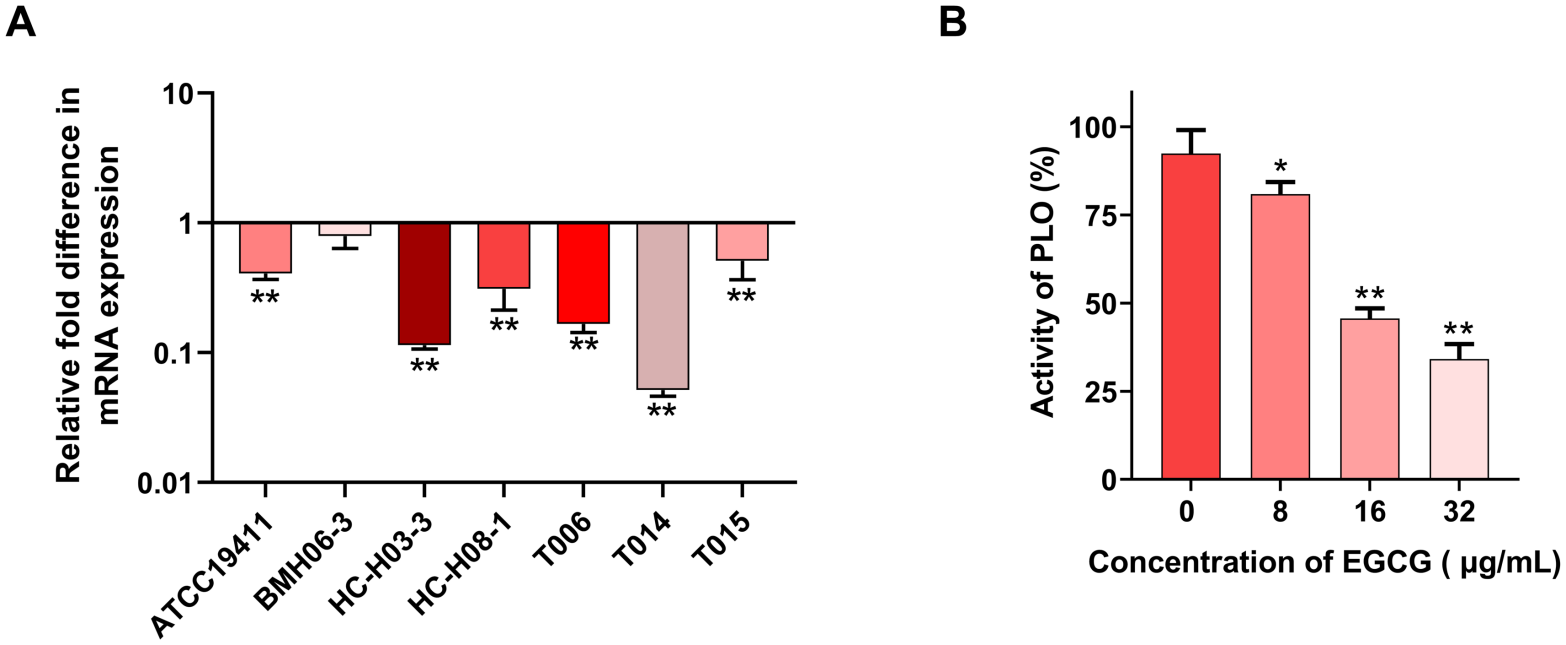
Inhibitory effects of EGCG on *plo* transcription and PLO-mediated hemolytic activity in *T. pyogenes*. **(A)** Relative mRNA expression of the *plo* gene after treatment with EGCG at 1/2 MIC. **(B)** Effects of different concentrations of EGCG on hemolytic activity. Statistical significance compared with the control group (untreated with EGCG) is indicated as *p < 0.05 and **p < 0.01. Data are presented as mean ± SD from three replicates.

To further assess the effect of EGCG on *plo* expression in *T. pyogenes*, additional isolates from dairy cattle were treated with EGCG at 1/2 MIC. The results showed that *plo* mRNA levels were reduced in all tested strains, including ATCC 19411. EGCG treatment significantly reduced the expression of the *plo* gene in six strains, with relative expression levels ranging from 0.05- to 0.51-fold those of the untreated controls (Supplementary Table 3, Figure 5A).

### 3.6 Inhibition of EGCG on the hemolytic activity of *Trueperella pyogenes*

The *in vitro* hemolysis assay was used to evaluate the hemolytic activity of *T. pyogenes*. As shown in Figure 5B, EGCG at subinhibitory concentrations significantly reduced the hemolytic activity of *T. pyogenes* ATCC 19411 in a concentration-dependent manner (*p* < 0.05).

### 3.7 Molecular docking and molecular dynamics analysis of EGCG–PLO interactions

**Figure 6:**
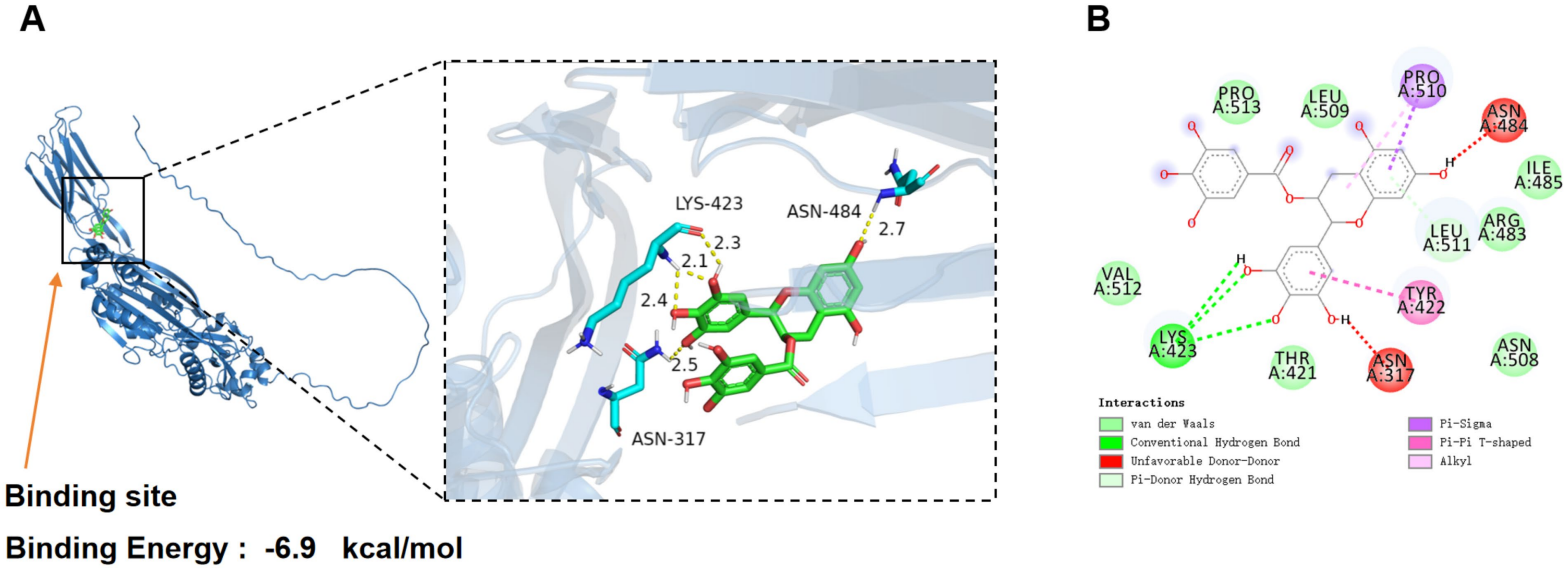
Molecular docking analysis of EGCG binding to PLO. **(A)** Three-dimensional structure of the stable PLO–EGCG complex. **(B)** Two-dimensional schematic diagram of the EGCG binding site on PLO.

Molecular docking analysis generated a binding model for the EGCG–PLO complex. The three-dimensional binding mode is illustrated in Figure 6A. The binding energy was −6.9 kcal/mol, supporting a favorable interaction between EGCG and PLO. The two-dimensional interaction diagram of the complex is shown in Figure 6B. EGCG bound to PLO through multiple non-covalent interactions. Hydrogen bonds with LYS423 contributed to the stabilization of the complex. In addition, EGCG formed *π*-π interactions with TYR422, π-*σ* interactions with PRO510, and a π-donor hydrogen bond with LEU511. Van der Waals contacts were observed with residues THR421, ARG483, ILE485, ASN508, LEU509, VAL512, and PRO513. EGCG exhibited a stable binding mode within the C-terminal region of PLO, where LYS423 and TYR422 served as key interacting residues.

**Figure 7:**
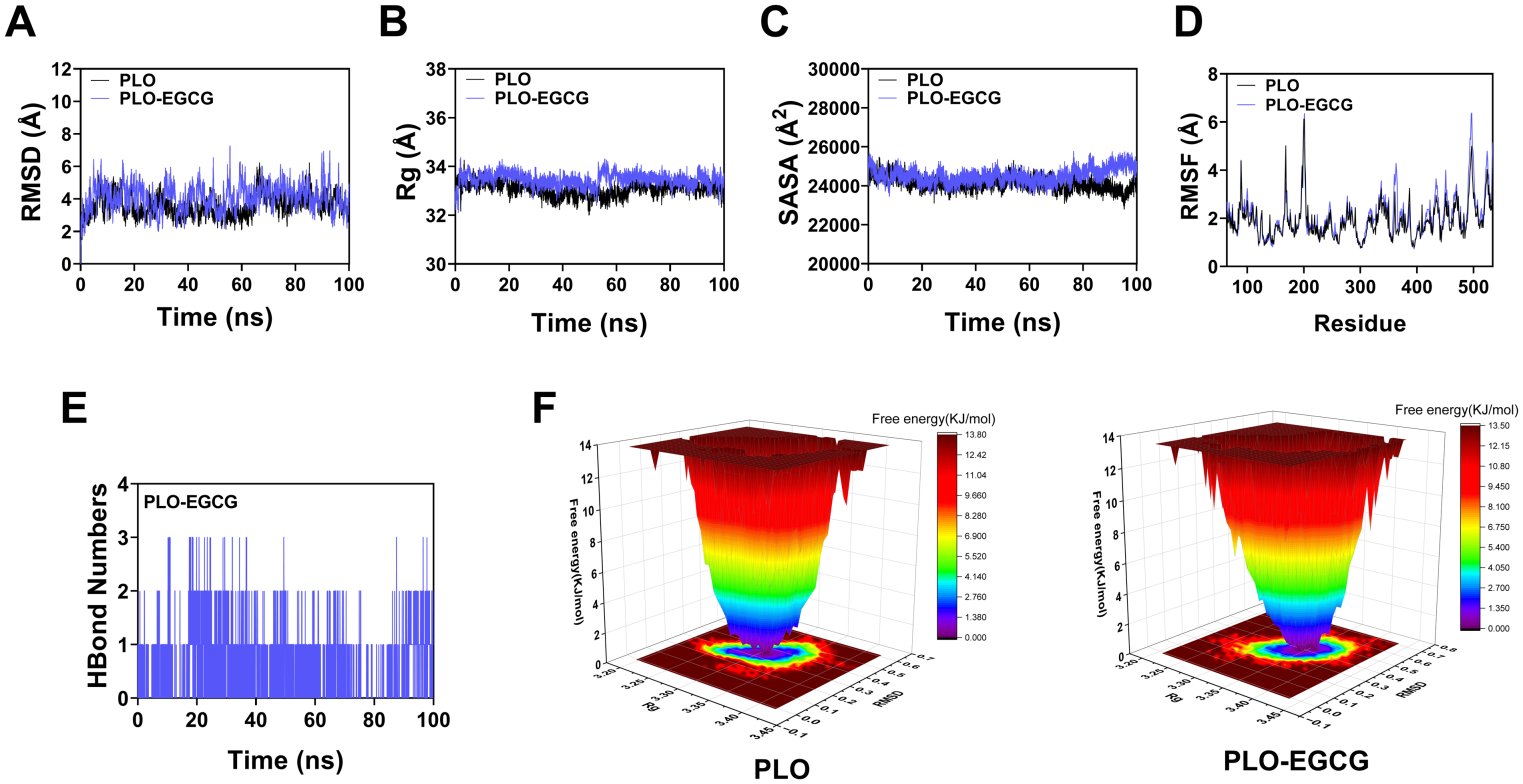
Molecular dynamics analysis of the PLO–EGCG complex. **(A)** RMSD of the PLO–EGCG complex. **(B)** Rg of the PLO–EGCG complex. **(C)** SASA of the PLO–EGCG complex. **(D)** RMSF profile of the PLO–EGCG complex. **(E)** Number of hydrogen bonds formed between PLO and EGCG over time. **(F)** FEL analysis of the PLO–EGCG complex. Blue regions corresponded to low-energy, stable conformations, and red regions represented higher-energy states.

The molecular dynamics simulation results showed that the RMSD of both the PLO and PLO–EGCG systems remained within 2–7 Å. These values indicate a moderate degree of conformational flexibility. Compared with the apo PLO system, the PLO–EGCG complex exhibited slight fluctuations but remained stable overall (Figure 7A). The Rg values of the two systems were comparable, ranging from 32–34 Å. EGCG binding did not markedly affect the compactness of PLO, and the overall folding state of the protein remained stable (Figure 7B). During the 0–100 ns simulation, the SASA of both systems showed only minor fluctuations (Figure 7C). EGCG binding had little effect on protein-solvent interactions, and the structural stability of PLO was maintained. The RMSF profile of the PLO–EGCG complex was comparable to that of apo PLO. A few residues showed slightly elevated fluctuations, but most regions remained stable (Figure 7D). Hydrogen bond analysis showed that the number of hydrogen bonds between EGCG and PLO fluctuated between 0 and 3, with approximately two bonds maintained for most of the simulation, indicating a stable interaction between EGCG and PLO (Figure 7E). Free energy landscapes (FELs) were constructed to characterize the conformational distribution and energy states of the systems. The FEL analysis further showed a dominant low-energy basin in both systems. This pattern indicated that EGCG binding did not disrupt the overall conformational stability of PLO (Figure 7F).

## 4 Discussion

*Trueperella pyogenes* is an opportunistic pathogen that affects both humans and animals, with infections reported in livestock, poultry, companion animals, and wildlife (1, 4, 25, 26). In recent years, increasing reports of human infections have raised concern (5, 6). Treatment of *T. pyogenes* infections is complicated by resistance gene-mediated antimicrobial resistance and biofilm formation (27). Therefore, the development of novel therapeutics against this pathogen is urgently required.

Natural compounds, particularly plant-derived phenolics, remain an important source of antibacterial candidates. Recent studies have shown that these compounds can affect several bacterial processes, including membrane integrity, biofilm formation, virulence-associated processes, and antimicrobial resistance mechanisms (28, 29). Consistently, compounds such as *α*-mangostin, myricetin, quercitrin, and luteolin have demonstrated antibacterial activity against pathogenic bacteria, especially Gram-positive pathogens, with relatively low toxicity and broad biological effects compared with conventional antibiotics (30–32). EGCG is one of the most widely reported flavonoids with antibacterial activity. In this study, we evaluated its antimicrobial efficacy against *T. pyogenes*. The *in vitro* findings suggest that EGCG inhibited the growth of *T. pyogenes* at concentrations ranging from 16 to 64 μg/mL. This observation is consistent with our previous study, which showed that luteolin also exhibited antibacterial activity against *T. pyogenes* (21). Notably, EGCG has higher water solubility than luteolin, which may favor its *in vivo* application. It has been shown that EGCG possesses antibacterial activity against both Gram-positive and Gram-negative bacteria, mainly through disruption of the bacterial cell wall and membrane, inhibition of intracellular proteins, DNA damage, and suppression of virulence factors (33, 34). We found that EGCG exhibits an antibacterial mechanism similar to that of luteolin (21). At subinhibitory concentrations, EGCG disrupts the cell wall of *T. pyogenes*, alters membrane permeability, and reduces intracellular protein and nucleic acid levels in the bacteria. These results suggest that the antibacterial activity of EGCG against *T. pyogenes* is primarily associated with disruption of bacterial cell structural integrity.

Biofilm-associated resistance compromises antibiotic efficacy in many chronic infections, and alternative agents may offer effective strategies for overcoming this limitation (35). However, only a limited number of naturally derived agents have been reported to inhibit biofilm formation in *T. pyogenes*, including luteolin and bacteriophage vB_EcoM-UFV13 (22, 36). EGCG is one of the earliest flavonoids investigated for its antibiofilm activity (37). It inhibits biofilm formation in a range of bacteria, including *E. coli*, *P. aeruginosa*, *Staphylococcus aureus*, *Enterococcus faecalis*, *Campylobacter jejuni*, *S. pneumoniae*, and *Streptococcus mutans* (35, 38). Knidel et al. (39) evaluated the antibiofilm activity of EGCG against nine *S. aureus* isolates and found that subinhibitory concentrations of EGCG inhibited biofilm formation. Similarly, our data show that subinhibitory concentrations of EGCG inhibit biofilm formation by *T. pyogenes* and that EGCG at 512 μg/mL can completely eradicate mature biofilms. This finding further supports the *in vitro* antibacterial activity of EGCG against *T. pyogenes*.

In a mouse infection model, EGCG exhibited *in vivo* antibacterial activity against *T. pyogenes*. Survival analysis revealed that a dose of 50 mg/kg conferred protection in infected mice, indicating that EGCG mitigated the severity of *T. pyogenes* infection. Liu et al. (40) reported the therapeutic efficacy of EGCG in an *S. aureus* SEA-induced injury model, in which EGCG at 25 and 50 mg/kg alleviated intestinal damage. This dose range is comparable to the effective dose observed in our mouse infection model. As a major immune organ, the spleen serves as an important indicator of host responses during infection (41). Accordingly, splenic index, bacterial load, and histopathological changes were assessed across groups to evaluate the therapeutic effect of EGCG in *T. pyogenes* infection. Although gentamicin treatment resulted in a lower splenic bacterial load than EGCG, the splenic index and histopathological findings indicated that EGCG provided comparable tissue protection. This suggests that the therapeutic effect of EGCG in *T. pyogenes* infection may not be solely attributable to its direct antibacterial activity but may also involve suppression of bacterial virulence and alleviation of splenic damage.

Hemolysins are key virulence factors in many pathogenic bacteria, and EGCG has been shown to inhibit hemolysin activity in *S. aureus*, *L. monocytogenes*, and *Aeromonas hydrophila* (18, 19, 42). PLO is the sole hemolysin of *T. pyogenes* and is considered a potential therapeutic target for anti-virulence strategies (4). Our results showed that EGCG at 1/2 MIC significantly downregulated *plo* transcription in *T. pyogenes*, while subinhibitory concentrations reduced the hemolytic activity of ATCC 19411. These findings suggest that transcriptional inhibition of *plo* may contribute to the EGCG-mediated reduction in hemolytic activity. Similar results have been reported in *Streptococcus suis*, where EGCG at MIC levels significantly reduced hemolytic activity and downregulated the expression of the hemolysin-encoding gene *sly* (43). PLO consists of four domains (D1–D4), with domain 4 (D4) located at the C-terminus. This domain mediates binding to cholesterol-rich cell membranes and anchors the PLO monomer to the membrane (44, 45). *In silico* analyses indicated that EGCG primarily bound to D4 of PLO (397–507 amino acids), with Lys423 and Tyr422 serving as key amino acid residues involved in EGCG–PLO binding (46). This suggests that EGCG may reduce the hemolytic activity of *T. pyogenes* by interfering with the interaction between PLO and erythrocyte membranes. Molecular dynamics simulations further demonstrated that the EGCG–PLO complex remained in a stable bound state. These results support PLO as a potential target of EGCG. The mechanism underlying the effects of EGCG on PLO requires further investigation in future studies.

There are still some limitations in this study. Although several bovine clinical isolates were included in the antimicrobial susceptibility and antibiofilm assays, most mechanistic experiments were conducted using the reference strain *T. pyogenes* ATCC 19411. Therefore, studies involving more isolates from different hosts and geographical origins are still needed to evaluate the broader applicability of EGCG against *T. pyogenes*. In addition, the interaction between EGCG and PLO was mainly explored by molecular docking and molecular dynamics simulations. Although these analyses suggested a stable EGCG–PLO interaction, direct experimental validation is still lacking. These gaps limit a more comprehensive understanding of the antibacterial and anti-virulence potential of EGCG against *T. pyogenes*. Future studies should address these issues by including more diverse clinical isolates and performing biophysical assays such as isothermal titration calorimetry, thermal shift assay, or cellular thermal shift assay, together with related functional experiments to further validate the EGCG–PLO interaction and clarify its role in the anti-virulence activity of EGCG.

## 5 Conclusion

In summary, this study demonstrated the antibacterial activity of EGCG against *T. pyogenes* both *in vitro* and *in vivo*. As shown in Supplementary Figure 4, EGCG acted through multiple mechanisms, including disruption of bacterial cell integrity, inhibition of biofilm formation, and attenuation of virulence and hemolytic activity. In addition, EGCG may reduce hemolytic activity by downregulating *plo* transcription and potentially interacting with PLO. These findings support the potential of EGCG as a therapeutic candidate for the prevention and treatment of *T. pyogenes* infections.

## Data Availability

The data supporting this study are available in the article and Supplementary material. The RNA-seq data have been deposited in the NCBI SRA database under BioProject accession number PRJNA1417744. Further inquiries can be directed to the corresponding authors upon reasonable request.
