# Supplementary material for: Antibacterial activity of epigallocatechin gallate against Trueperella pyogenes and its potential effect on pyolysin
Source: Front Vet Sci. 2026 Jul 8;13:1889070. doi: 10.3389/fvets.2026.1889070 (PMC13388063; doi:10.3389/fvets.2026.1889070)
Supplement: Supplementary file 1 [file Data_Sheet_1.DOCX]

Supplementary Material

# Supplementary Figures and Tables

## Supplementary Figures


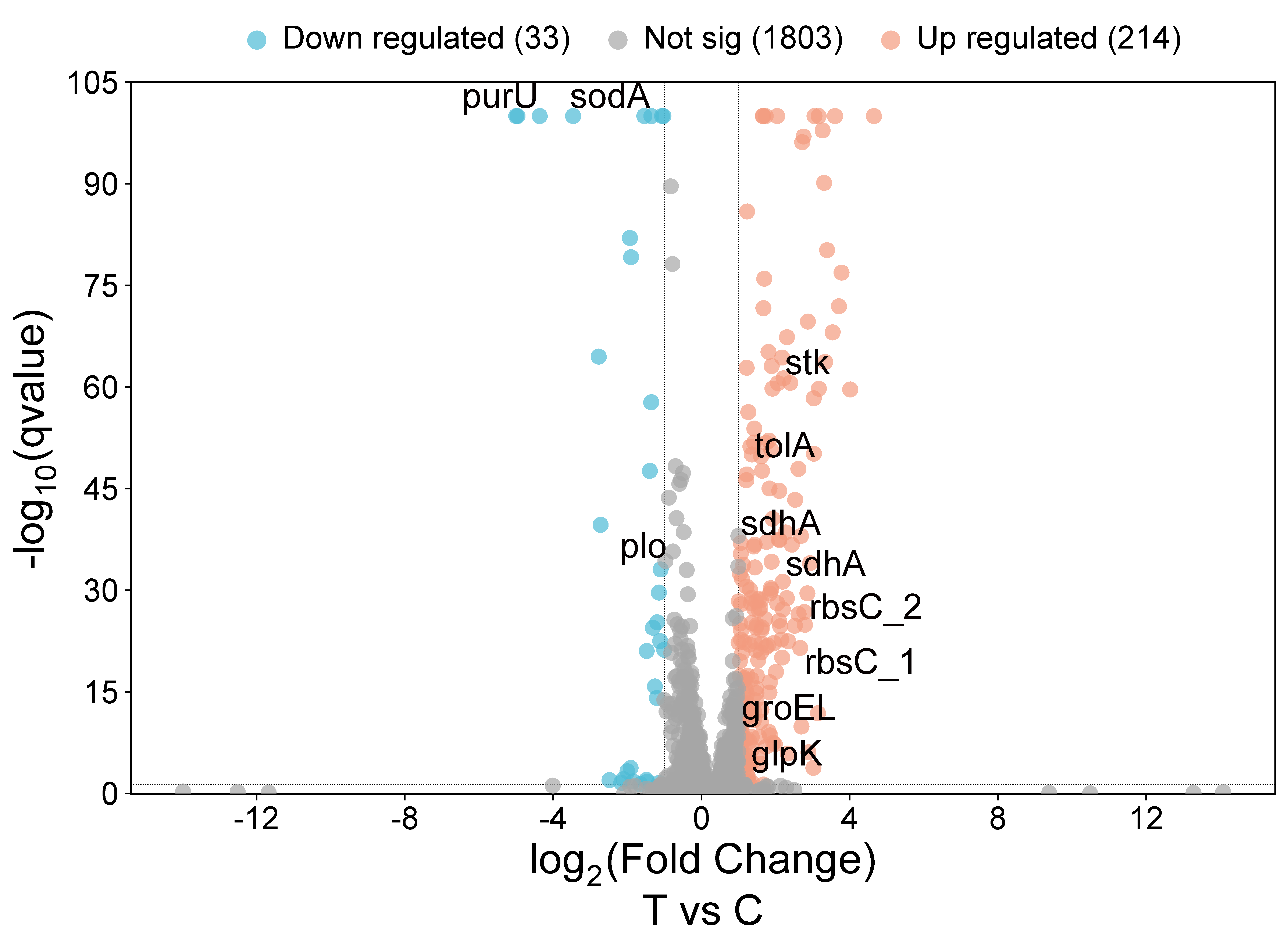


**Supplementary Figure 1. Volcano plot showing significant DEGs between the untreated control group and the 1/2 MIC EGCG-treated group.**

**
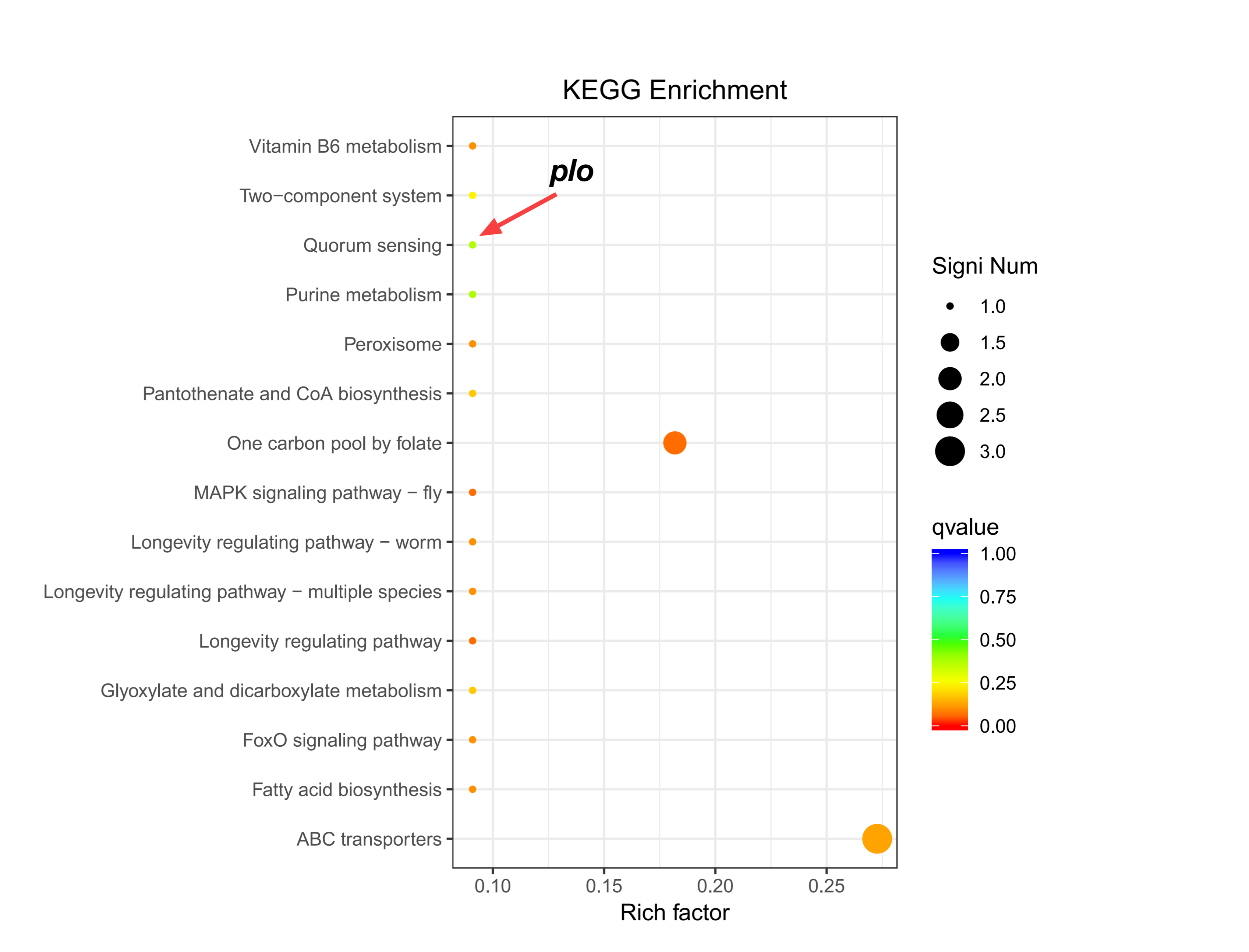
**

**Supplementary Figure 2. KEGG enrichment analysis of significantly downregulated DEGs in the 1/2 MIC EGCG-treated group compared with the untreated control group.**


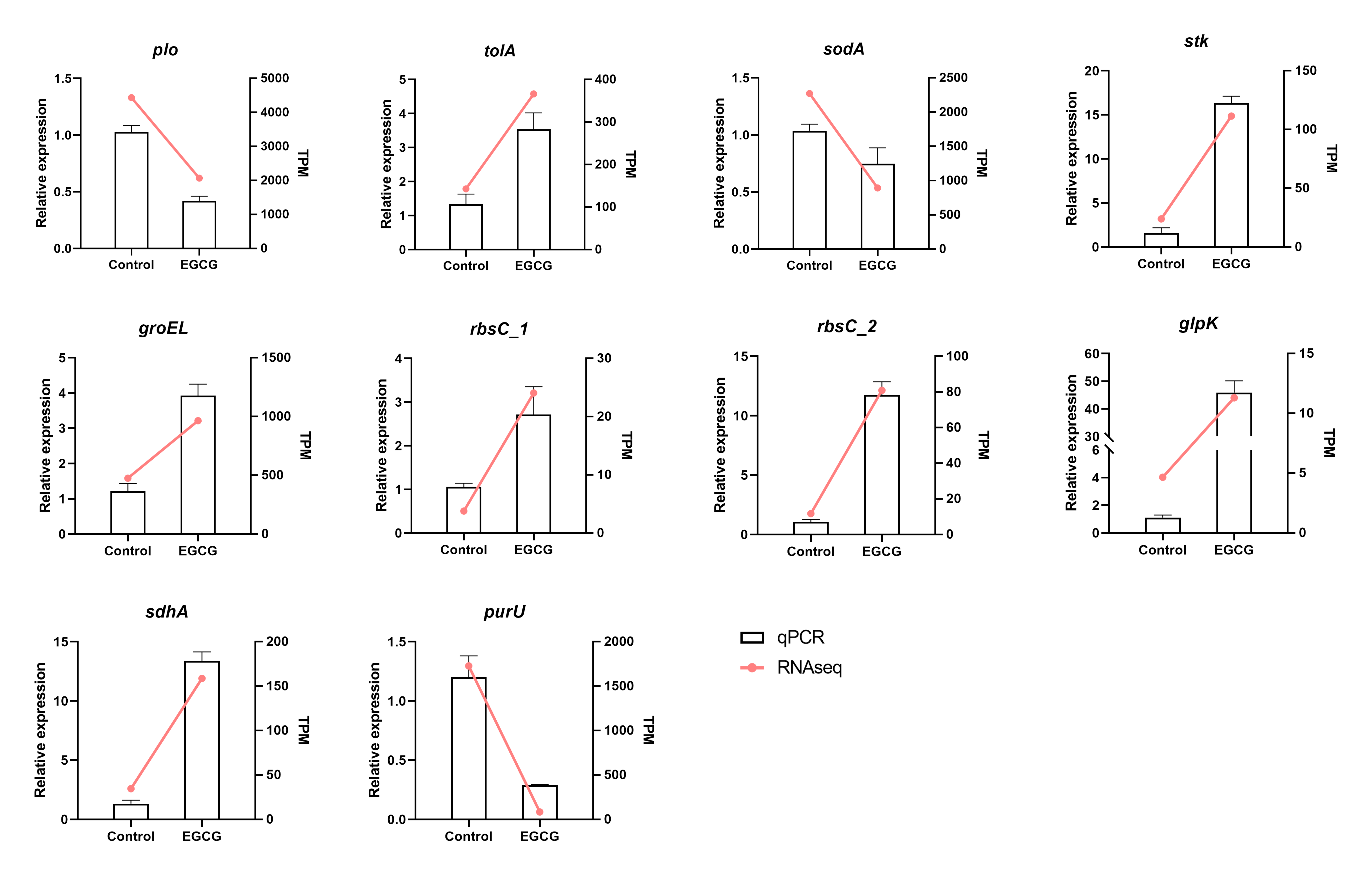


**Supplementary Figure 3. qPCR validation of selected DEGs from the transcriptome analysis.**


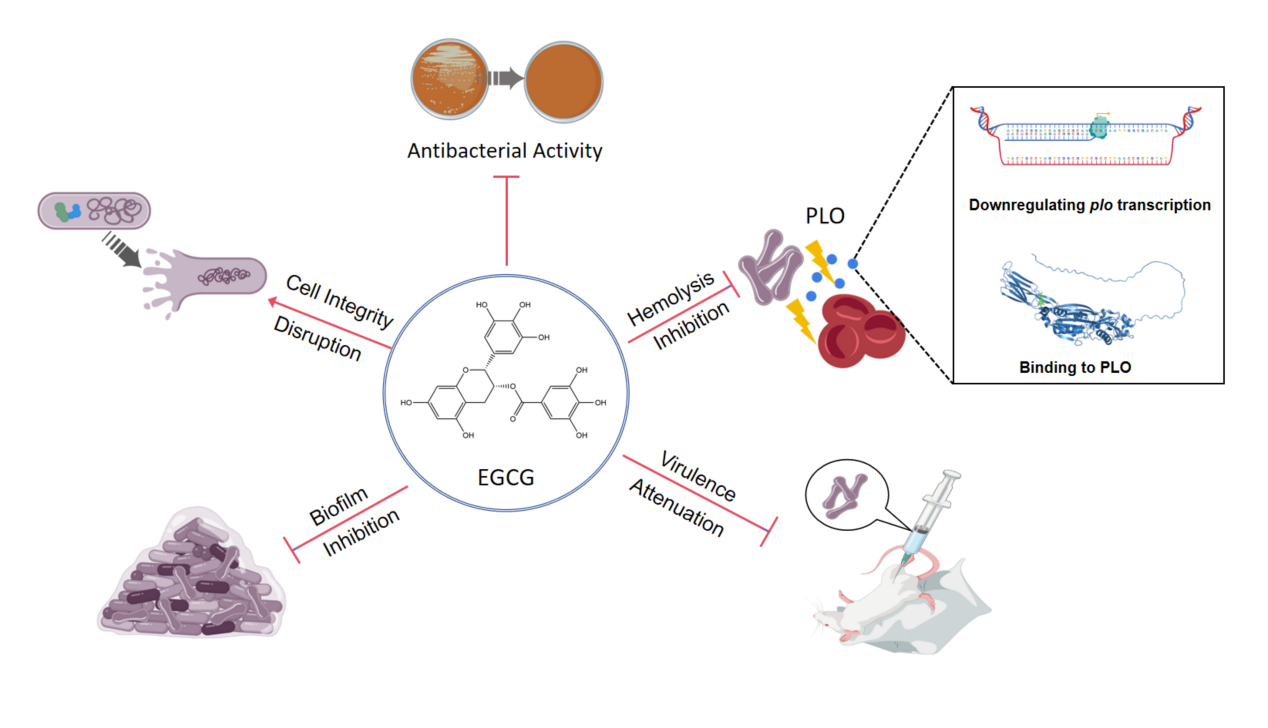


**Supplementary Figure 4. Multiple antibacterial actions of EGCG against *Trueperella pyogenes*.**

The graphical abstract was generated using the Generic Diagramming Platform (BioGDP).

## Supplementary Tables

**Supplementary Table 1. *Trueperella pyogenes* strains and source information**

| No. | Strains | Source |
| --- | --- | --- |
| 1 | ATCC19411 | reference strain |
| 2 | BMH06-3 | bovine endometritis |
| 3 | HC-H03-3 | bovine endometritis |
| 4 | HC-H08-1 | bovine endometritis |
| 5 | T006 | bovine mastitis |
| 6 | T014 | bovine mastitis |
| 7 | T015 | bovine mastitis |

**Supplementary Table 2. Primer sequences for qPCR used in this study**

| Primer name | Nucleotide sequence (5'–3') | Product size (bp) |
| --- | --- | --- |
| *16S rRNA*-F | ATGCAACGCGAAGAACCTTACC | 127 |
| *16S rRNA*-R | TTAACCCAACATCTCACGACAC |  |
| *plo*-F | TTGCCTCCAGTTGACGCTTTGAC | 115 |
| *plo*-R | GCCTTCTCGACGGTTGGATTCAG |  |
| *tolA*-F | TCAAGCCTGCTCCAGACAAG | 105 |
| *tolA*-R | AAGCTGTCATCATGCCGTCA |  |
| *sodA*-F | CCAGTTGGCACTTACCCGAT | 107 |
| *sodA*-R | AAGACATTCCACACGGCCTT |  |
| *stk*-F | GGAGTGGTTCAAGCGCTACT | 167 |
| *stk*-R | CTTCGAAGATCTTGCGCTGC |  |
| *groEL*-F | AACCGCTGAGGACATCAAGG | 86 |
| *groEL*-R | GGAGCTTCTCGCGATCGTAA |  |
| *rbsC_1*-F | GCGTCTATATGCTGGCAGGT | 154 |
| *rbsC_1*-R | GCTTTACCACCACGAAGGGA |  |
| *rbsC_2*-F | GCTTGTTATTTCCGACGGCC | 138 |
| *rbsC_2*-R | GTGCGGGTGAGGATAAACCA |  |
| *glpK*-F | CATTCCAGACAGCCGAGGTT | 136 |
| *glpK*-R | ACGTCAACGCCGAGGATATC |  |
| *sdhA*-F | CCGTCTACTTCCAGTCCACG | 167 |
| *sdhA*-R | CGACTCCGACATGAGGATCG |  |
| *purU*-F | TCCACCATTCTTTCCTGCCC | 171 |
| *purU*-R | CGAGCTCGTCATCATCGTGA |  |

**Supplementary Table 3. Fold changes in mRNA expression of *T. pyogenes***

**following treatment with 1/2 MIC EGCG**

| No. | Strains | Fold changes in mRNA expression |
| --- | --- | --- |
| 1 | ATCC19411 | 0.41 |
| 2 | BMH06-3 | 0.79 |
| 3 | HC-H03-3 | 0.11 |
| 4 | HC-H08-1 | 0.31 |
| 5 | T006 | 0.17 |
| 6 | T014 | 0.05 |
| 7 | T015 | 0.51 |
